# Potential risk for bacterial contamination in conventional reused ventilator systems and disposable closed ventilator-suction systems

**DOI:** 10.1371/journal.pone.0194246

**Published:** 2018-03-16

**Authors:** Ya-Chi Li, Hui-Ling Lin, Fang-Chun Liao, Sing-Siang Wang, Hsiu-Chu Chang, Hung-Fu Hsu, Sue-Hsien Chen, Gwo-Hwa Wan

**Affiliations:** 1 Department of Respiratory Therapy, Chang Gung Memorial Hospital, Keelung, Taiwan; 2 Department of Respiratory Therapy, College of Medicine, Chang Gung University, Taoyuan, Taiwan; 3 Department of Respiratory Therapy, Chang Gung Memorial Hospital, Chiayi, Taiwan; 4 Department of Respiratory Care, Chang Gung University of Science and Technology, Chiayi, Taiwan; 5 Department of Industrial Design, College of Management, Chang Gung University, Taoyuan, Taiwan; 6 Department of Nursing, Chang Gung Memorial Hospital, Keelung, Taiwan; 7 Department of Obstetrics and Gynaecology, Taipei Chang Gung Memorial Hospital, Taipei, Taiwan; National Yang-Ming University, TAIWAN

## Abstract

**Background:**

Few studies have investigated the difference in bacterial contamination between conventional reused ventilator systems and disposable closed ventilator-suction systems. The aim of this study was to investigate the bacterial contamination rates of the reused and disposable ventilator systems, and the association between system disconnection and bacterial contamination of ventilator systems.

**Methods:**

The enrolled intubated and mechanically ventilated patients used a conventional reused ventilator system and a disposable closed ventilator-suction system, respectively, for a week; specimens were then collected from the ventilator circuit systems to evaluate human and environmental bacterial contamination. The sputum specimens from patients were also analyzed in this study.

**Results:**

The detection rate of bacteria in the conventional reused ventilator system was substantially higher than that in the disposable ventilator system. The inspiratory and expiratory limbs of the disposable closed ventilator-suction system had higher bacterial concentrations than the conventional reused ventilator system. The bacterial concentration in the heated humidifier of the reused ventilator system was significantly higher than that in the disposable ventilator system. Positive associations existed among the bacterial concentrations at different locations in the reused and disposable ventilator systems, respectively. The predominant bacteria identified in the reused and disposable ventilator systems included *Acinetobacter spp*., *Bacillus cereus*, *Elizabethkingia spp*., *Pseudomonas spp*., and *Stenotrophomonas (Xan) maltophilia*.

**Conclusions:**

Both the reused and disposable ventilator systems had high bacterial contamination rates after one week of use. Disconnection of the ventilator systems should be avoided during system operation to decrease the risks of environmental pollution and human exposure, especially for the disposable ventilator system.

**Trial registration:**

ClinicalTrials.gov PRS / NCT03359148

## Introduction

In clinical practice, patients with acute respiratory failure or severe diseases must use ventilators for life support [1, 2]. The American Association for Respiratory Care clinical practice guidelines state that the ventilator system used for critically ill patients does not need to be changed daily for the purpose of infection control [3]; however, the maximum time until which a system can be continually used safely remains unknown. The correlation between ventilator-associated pneumonia (VAP) and heated or unheated humidifiers, the type of humidifier, humidifier water refill method, and removal of condensate water from the system is still uncertain. Currently, research into ventilator system contamination is limited.

Eighty percent of the condensate samples from the ventilator circuits were contaminated with a median bacterial concentration of 2x10^5^ organisms/mL when the circuit systems were used for 24 h [4]. The bacteria isolated from the circuit condensates were correlated with those isolated from the patients’ sputum, suggesting that the patients’ oropharyngeal flora were the primary source of circuit colonization [4]. Additionally, when the frequency of ventilator system replacement was the same between systems, the contamination rates of disposable ventilator systems could be higher than those of conventional ventilator systems [5]. It is suggested that ventilator circuits with heated humidification may be changed every 48 h in adult patients [6]. However, the incidence of ventilator associated pneumonia when ventilator circuits were changed every 2 or 3 days did not differ from that when ventilator circuits were changed every 7 days in adult and neonatal intensive care units [7,8]. The American Association for Respiratory Care (AARC) guidelines suggest that changing ventilator circuits routinely is not necessary unless ventilator circuits have visibly soiled or malfunction [9]. Previous studies indicate that bacteria cultured from the ventilator systems included *Pseudomonas aeruginosa*, *Haemophilus* spp., *Enterobacteriaceae*, methicillin-sensitive *Staphylococcus aureus*, and methicillin-resistant *staphylococcus aureus* [4,10].

The sputum culture from an open suction system had a higher bacterial concentration than a closed suction system [11], and an open suction system was associated with a higher risk of acquiring VAP [12,13]. However, no difference in the VAP rate was found between closed and open suction systems [14,15]. Although the closed suction system is designed for reducing costs and cross contamination, the unit price is currently still higher than an open suction tube, resulting in limited use. To the best of our knowledge, previous studies have rarely evaluated bacterial contamination inside ventilator systems, including conventional reused ventilator-open suction systems and disposable closed ventilator-suction systems.

Therefore, the aim of this study was to investigate the bacterial contamination rates of the reused and disposable ventilator systems after one week of continuous use, and the association between system disconnection and bacterial contamination of ventilator systems.

## Methods

### Study location and patients

**Fig 1:**
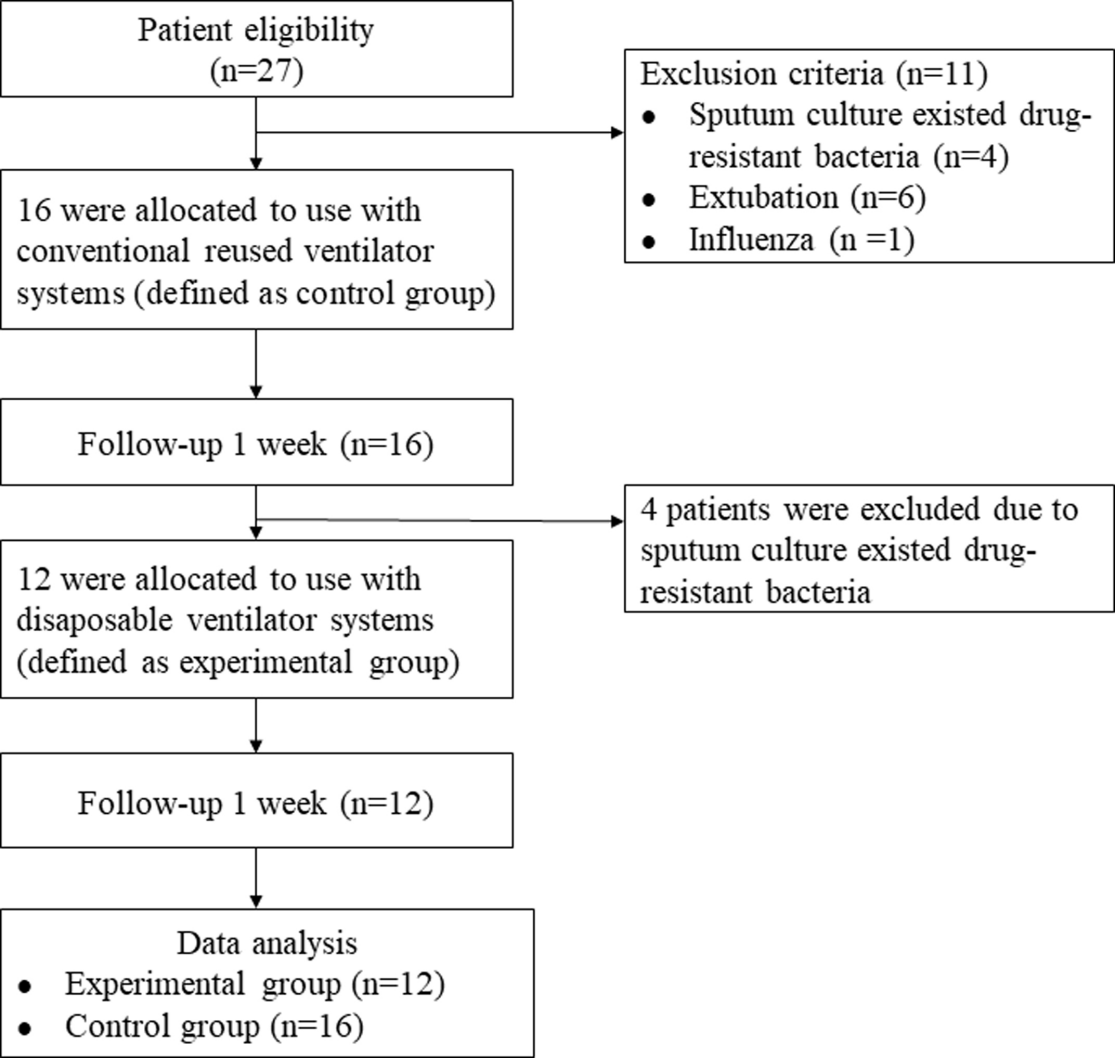
Diagram depicting the enrollment and follow-up of the study participants.

This study was performed from April 2015 to July 2016. The orally intubated and mechanically ventilated patients from the Intensive Care Unit of Keelung Chang Gung Memorial Hospital in Taiwan were enrolled in this study. For safety of the researchers, all the drug-resistant bacteria infected patients were excluded from the study. In this study, the sputum culture results of the patients only included three drug-resistant bacteria (*Acinetobacter baumannii*, *Klebsiella pneumonia*, and *Mycobacterium tuberculosis*). The exclusion criteria included extubation and if the sputum culture results of the patients indicated the presence of drug-resistant bacteria (including *Acinetobacter baumannii*, *Klebsiella pneumonia*, *Mycobacterium tuberculosis*). A flowchart depicting the enrollment and follow-up of the study participants is shown as Fig 1. Every subject used the reused ventilator system first and then used the disposable ventilator system because of cost consideration. The study design was an observational study. Each patient’s medical history was recorded, including sex, age, admission and intubation date, primary diagnosis, ventilator records, and sputum analysis. The study was approved by the Institutional Review Board of Chang Gung Memorial Hospital (IRB Case Number: 101-5140B). The purpose and process of the study were explained to each patient and their family, and an informed consent form was signed by patient’s family before study admission.

### Conventional reused ventilator system and disposable ventilator system

The experimental study group was assigned to a disposable ventilator system (Fisher & Paykel Healthcare Limited, Auckland, New Zealand) combined with an auto-filled heated humidifier (HH), a closed suction catheter (Pacific Hospital Supply Co. Ltd., Taipei, Taiwan), and a closed aerosol therapy procedure with a valved T-adaptor (Galemed Corp., I-Lan, Taiwan). The control study group was assigned to a conventional reused ventilator system (Galemed Corp., I-Lan, Taiwan), combined with a manually filled HH, an open suction catheter (Symphon Medical Technology Co. Ltd., Taipei, Taiwan), and a conventional aerosol therapy procedure. After using the reused ventilator systems continuously for 1 week, patients were switched to the disposable ventilator systems according to the study protocol of ventilator circuit change. This study was designed to change the ventilator circuit system after one week of continuous use according to the routine guideline of clinical practices in Taiwanese hospitals [16].

### Bacterial sampling and analysis

**Fig 2:**
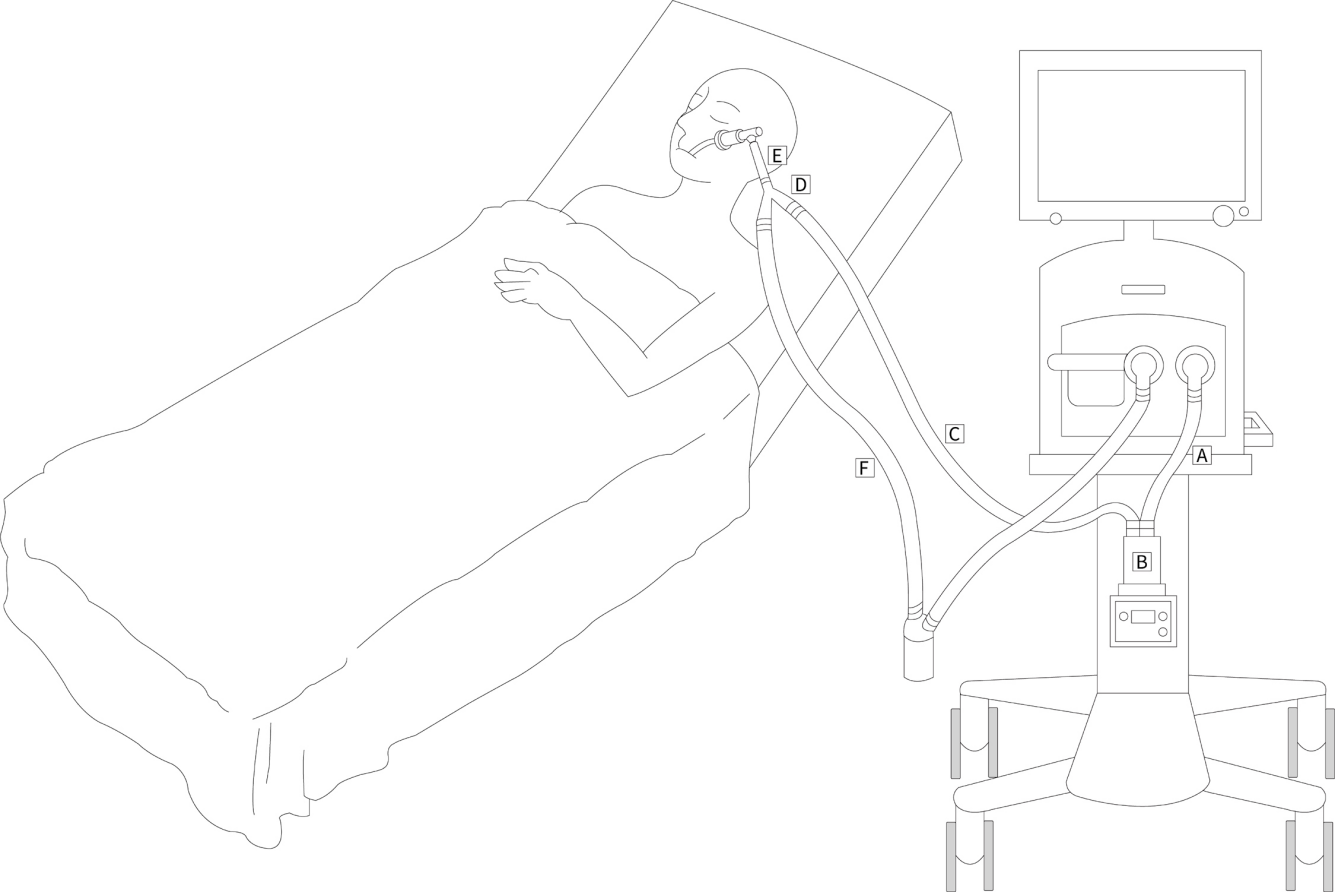
Diagram of a ventilator system including the inlet tube of the HH (A), the HH (B), inspiratory limb (C), Y-adapter (D), 15-cm corrugated tube (E), and the expiratory limb (F).

The ventilator system, including the inlet tube of the HH, the HH, Y-adapter, 15-cm corrugated tube, and the inspiratory/expiratory limbs (Fig 2), was removed after a week of use; the interior of tubes was washed with 15 mL of sterile distilled water and the water samples were collected from the HHs immediately. The samples were diluted and inoculated on culture plates at 35 ± 1°C for 48 ± 2 h. All bacterial species were identified biochemically. In this study, the primary and secondary outcomes measured in the reused and disposable ventilator systems were the bacterial concentration and bacterial detection rate, respectively.

### Statistical analysis

Data were analyzed using SPSS version 21.0 (IBM Corp., Armonk, NY, USA). All figures were constructed with GraphPad Prism version 6.0 (GraphPad Software, San Diego, CA, USA). A two-sided *P* value of < 0.05 was considered statistically significant. In a Wilcoxon signed-rank test study, sample sizes of 16 and 12 were obtained from the reused ventilator system and disposable ventilator system that zero-mean difference discrimination was tested. The total sample of 28 ventilator systems had an 83% power to detect the mean difference versus the alternative of zero-mean difference discrimination using a t test with a 0.05 significance level. A chi-square test was used to compare the detection rates between the reused and disposable ventilator systems. The bacterial concentration distributions of the reused and disposable ventilator systems were compared using the Wilcoxon signed-rank test. Spearman correlation analysis was applied to identify the relationship between two continuous variables with non-normally distributed data.

## Results

We recruited 27 intubated and mechanically ventilated patients; however, 11 patients were excluded since their sputum culture results indicated the presence of drug-resistant bacteria (n = 4; including *Mycobacterium tuberculosis*, multi-drug resistant *Acinetobacter baumannii*, pandrug-resistant *Acinetobacter baumannii*, and carbapenem-resistant *Acinetobacter baumannii*), as well as due to early extubation (n = 6) and the presence of influenza infection (n = 1). Therefore, 16 patients (10 men, 6 women; aged 20–91 years) were included from the final analysis. Patients had been on a mechanical ventilator for 17–46 days, and had a primary diagnosis of sepsis, septic shock, cardiac arrest, pneumonia (community-acquired or hospital-acquired), chronic obstructive pulmonary disease, respiratory failure, acute respiratory distress syndrome, or lung contusion (Table 1).

**Table 1:** Personal characteristics of study subjects.

ID	Sex	Age	Ventilation day	Major diagnosis
1	F	78	17	Severe sepsis, hypercapnic respiratory failure, pneumonia
2	F	81	29	Cardiac arrest, hypoxemic encephalopathy
3	F	83	29	Acute respiratory failure, fever
4	M	79	20	HCAP, respiratory failure
5	M	80	26	HCAP with hypercapnic respiratory failure, COPD with acute exacerbation
6	M	81	29	COPD with acute exacerbation, VAP
7	F	83	24	CAP with respiratory failure
8	M	63	20	Septic shock
9	F	71	23	Mixed type respiratory failure, VAP
10	M	78	21	Mixed type respiratory failure, cardiac arrest
11	M	70	21	Nosocomial pneumonia
12	M	69	21	Influenza A pneumonia with acute respiratory distress syndrome
13	M	91	33	HAP
14	M	49	36	HCAP
15	M	20	46	Bilateral lung contusion
16	F	42	22	HAP with acute respiratory distress syndrome

HCAP, healthcare-associated pneumonia; COPD, chronic obstructive pulmonary disease; VAP, ventilator-associated pneumonia; CAP, community-acquired pneumonia; HAP, hospital-acquired pneumonia.

Bacterial samples from 16 patients were taken from both ventilator systems. Bacterial analysis results from four patients on the disposable ventilator system were excluded since the sputum culture indicated the presence of multi-drug resistant *Acinetobacter baumannii*. Bacterial contamination of the conventional reused ventilator system (94.8%) was significantly higher than that of the disposable ventilator system (81.9%; *P* < 0.01; Table 2 and S1 File). The locations with the highest contamination rates were the inspiratory limb (100%), Y-adapter (100%), 15-cm corrugated tube (100%), and expiratory limb (100%) in both systems. The detection rates of bacteria significantly differed in different locations of the conventional reused ventilator system (*P* = 0.02) and the disposable ventilator system (*P* < 0.01). There was a significant difference in the bacterial concentration distribution between different sampling locations in both systems. The bacterial concentration in the expiratory limb (1.47 × 10^7^ colony forming units [CFU]/mL) of the reused ventilator system was significantly higher than the other locations on the ventilator system, followed by the 15-cm corrugated tube (7.43 × 10^6^ CFU/mL), and the inlet tube of the HH, which had the lowest concentration distribution (20 CFU/mL). The expiratory limb of the disposable ventilator system (1.28 × 10^8^ CFU/mL) had the highest bacterial concentration, followed by the inspiratory limb (2.56 × 10^7^ CFU/mL), with the inlet tube of the HH showing seldom bacterial growth (0‒10 CFU/mL). Additionally, bacterial concentrations in the inlet tube of the HH (*P* = 0.021) and the HH (*P* = 0.004) of the reused ventilator system were higher than those of the disposable ventilator system. However, the bacterial concentrations in the inspiratory limb (*P* = 0.002) and expiratory limb (*P* = 0.004) of the disposable ventilator system were significantly higher than those in the inspiratory and expiratory limbs of the reused ventilator system.

**Table 2:** Detection rates and concentrations of bacteria detected at different locations of reused and disposable ventilator systems.

Location	Detection rate (n, %)			Concentration (CFU/mL)		
	Reused system (n = 16)	Disposable system (n = 12)	*P* value	Reused system (n = 16)	Disposable system (n = 12)	*P* value
Total	91 (94.8%)	59 (81.9%)	< 0.01	1.98 × 10^6^ (5.98 × 10^5^–1.43 × 10^7^)	2.41 × 10^6^ (1.9 × 10^5^–2.41 × 10^7^)	0.616
IHH	12 (75%)	2 (16.7%)	< 0.01	20.00 (2.5–75.00)^#^^†^^‡^^§^^@^^¶^	0 (0–10)^#^^†^^‡^^§^^@^^¶^	0.021
HH	15 (93.8%)	9 (75%)	0.168	1.96 × 10^6^ (8.25 × 10^5^–5.13 × 10^6^)^*^^¶^	2.08 × 10^5^ (2.50 × 10^3^–7.05 × 10^5^)^*^^‡^^§^^@^^¶^	0.004
IL	16 (100%)	12 (100%)	—	2.69 × 10^6^ (1.53 × 10^6^–1.48 × 10^7^)^*^^§^^@^^¶^	2.56 × 10^7^ (1.29 × 10^7^–1.42 × 10^8^)^*^^†^^§^	0.002
Y-adapter	16 (100%)	12 (100%)	—	1.25 × 10^6^ (6.13 × 10^5^–1.72 × 10^6^)^*^^‡^^@^^¶^	1.51 × 10^6^ (1.12 × 10^6^–4.08 × 10^6^)^*^^†^^‡^	0.117
15-cm tube	16 (100%)	12 (100%)	—	7.43 × 10^6^ (1.76 × 10^6^–4.79 × 10^7^)^*^^§^	1.82 × 10^7^ (5.19 × 10^6^–2.43 × 10^7^)^*^^†^	0.099
EL	16 (100%)	12 (100%)	—	1.47 × 10^7^ (8.25 × 10^6^–1.93 × 10^7^)^*^^†^^§^	1.28 × 10^8^ (1.35 × 10^7^–2.55 × 10^8^)^*^^†^	0.004
*P* value	0.02	< 0.01		< 0.01	< 0.01	

IHH: IHH, inlet tube of the HH; HH, heated humidifier; IL, inspiratory limb; EL, expiratory limb.

^#^: Data was presented as median (min–max).

*: compared to IHH;

†: compared to HH;

^‡^: compared to IL;

^§^: compared to Y-adapter;

^@^: compared to 15-cm tube;

^¶^: compared to EL.

The average disconnection frequency of the reused ventilator system (89.25 times/week) was significantly higher than that of the disposable ventilator system (11.75 times/week; *P* < 0.01; S1 File). However, there was no significant association between the ventilator disconnection frequency and bacterial concentration at different locations on the reused and disposable ventilator systems, respectively (Table 3). In the reused ventilator system, positive associations existed among the bacterial concentrations of the Y-adapter, 15-cm corrugated tube, and expiratory limb (r_s_ = 0.549–0.677, *P* < 0.05). Also, the bacterial concentration of the inspiratory limb was associated with that of the HH (r_s_ = 0.603, *P* = 0.013). With the disposable ventilator system, bacterial growth on the Y-adapter was positively associated with that on the 15-cm corrugated tube (r_s_ = 0.797, *P* < 0.01), HH (r_s_ = 0.592, *P* = 0.043), and expiratory limb (r_s_ = 0.767, *P* < 0.01). Additionally, the bacterial concentration of the 15-cm corrugated tube was positively associated with that of the expiratory limb (r_s_ = 0.697, *P* = 0.012).

**Table 3:** Associations of disconnection frequency and bacterial concentrations at different locations of reused and disposable ventilator systems.

	(1)	(2)	(3)	(4)	(5)	(6)	(7)
**Reused ventilator system**							
(1) Disconnection frequency	1						
(2) IHH	0.182	1					
(3) HH	0.185	0.35	1				
(4) IL	0.243	0.251	0.603*	1			
(5) Y-adapter	0.027	-0.159	0.193	0.353	1		
(6) 15-cm tube	0.094	-0.028	0.374	0.456	0.677**	1	
(7) EL	-0.223	-0.01	0.096	0.318	0.549*	0.571*	1
**Disposable ventilator system**							
(1) Disconnection frequency	1						
(2) IHH	-0.3	1					
(3) HH	0.106	-0.406	1				
(4) IL	-0.347	-0.275	0.346	1			
(5) Y-adapter	-0.098	-0.134	0.592*	0.445	1		
(6) 15-cm tube	0.306	-0.005	0.373	0.336	0.797**	1	
(7) EL	-0.137	-0.027	0.233	0.572	0.767**	0.697*	1

IHH, inlet tube of the HH; HH, heated humidifier; IL, inspiratory limb; EL, expiratory limb.

*: *P* < 0.05

**: *P* < 0.01.

The predominant bacteria cultured in sputum specimens from the mechanically ventilated patients included *Pseudomonas spp*. (22.22%), *Acinetobacter spp*. (16.67%), and *Klebsiella spp*. (16.67%), all of which are gram-negative bacteria (Table 4). Gram-negative bacteria were also frequently present in the conventional reused ventilator system (86.79%) and disposable ventilator system (82.5%). The isolation rates of *Stenotrophomonas (Xan) maltophilia* were the highest, followed by *Acinetobacter spp*. and *Pseudomonas spp*., in both systems. Additionally, both ventilator systems contained an average of two common environmental bacterial species.

**Table 4:** Bacterial species distributions of sputum specimens, reused and disposable ventilator systems.

Species	Sputum specimen		Reused ventilator system		Disposable ventilator system	
	(n = 16)		(n = 16)		(n = 16)	
**Gram-positive bacteria**	**2**	**(11.12%)**	**7**	**(13.21%)**	**7**	**(17.5%)**
*Bacillus cereus*	0	(0%)	2	(3.77%)	4	(10%)
*Corynebacterium spp*.	0	(0%)	3	(5.66%)	1	(2.5%)
*Enterococcus spp*.	1	(5.55%)	1	(1.89%)	1	(2.5%)
*Staphylococcus spp*.	1	(5.55%)	1	(1.89%)	1	(2.5%)
**Gram-negative bacteria**	**16**	**(88.88%)**	**46**	**(86.79%)**	**33**	**(82.5%)**
*Achromobacter spp*.	0	(0%)	0	(0%)	1	(2.5%)
*Acinetobacter spp*.	3	(16.67%)	14	(26.42%)	6	(15%)
*Bergeyella spp*.	0	(0%)	1	(1.89%)	0	(0%)
*Burkholderia spp*.	0	(0%)	3	(5.66%)	1	(2.5%)
*Chryseobacterium spp*.	0	(0%)	1	(1.89%)	1	(2.5%)
*Citrobacter spp*.	1	(5.55%)	0	(0%)	0	(0%)
*Delftia spp*.	0	(0%)	1	(1.89%)	1	(2.5%)
*Elizabethkingia spp*.	1	(5.55%)	5	(9.43%)	3	(7.5%)
*Escherichia spp*.	1	(5.55%)	0	(0%)	0	(0%)
*Glucose non-fermenting G(-) bacilli*	0	(0%)	0	(0%)	1	(2.5%)
*Haemophilus spp*.	1	(5.55%)	0	(0%)	0	(0%)
*Klebsiella spp*.	3	(16.67%)	0	(0%)	1	(2.5%)
*Moraxella spp*.	1	(5.55%)	0	(0%)	0	(0%)
*Pseudomonas spp*.	4	(22.22%)	6	(11.32%)	4	(10%)
*Ralstonia spp*.	0	(0%)	2	(3.77%)	1	(2.5%)
*Serratia spp*.	0	(0%)	1	(1.89%)	1	(2.5%)
*Sphingomonas spp*.	0	(0%)	2	(3.77%)	1	(2.5%)
*Stenotrophomonas (Xan) maltophilia*	1	(5.55%)	10	(18.88%)	11	(27.5%)

Data were presented as n (%).

## Discussion

To date, few studies have evaluated the bacterial contamination of ventilator systems for critical patients, especially with regard to the disposable ventilator system. In this study, the inlet tube of the HH and the HH of the reused ventilator system contained a greater bacterial concentration than the disposable ventilator system. The cause of this contamination may potentially be related to a synergistic effect of the contamination in the inspiratory limb and the repetitive disconnection for refilling distilled water into the HH of the reused ventilator system.

However, the bacterial concentrations on the inspiratory limb, Y-adapter, 15-cm corrugated tube, and the expiratory limb of the disposable ventilator system were higher than on those of the reused ventilator system. In this study, the average disconnection frequency of the disposable ventilator system was much lower than that of the reused ventilator system. The cause of disconnection of the disposable ventilator systems was mainly daily care of endotracheal tubes in patients. For reused ventilator systems, the causes of disconnection of the systems included filling sterile distilled water into HHs, and performing open suction technique and conventional aerosol therapy. Thus, the differences of bacterial contamination between the reused ventilator circuit system and the disposable ventilator circuit system possibly came from the type of suction systems used in the patients. Additionally, the study indicates that the bacterial growth on the Y-adapter was positively associated with that on the 15-cm corrugated tube, HH, and expiratory limb in the disposable ventilator system. Also, a positive association between the bacterial concentrations of the expiratory limb and 15-cm corrugated tube was found in the disposable ventilator system. Thus, we speculate that the unfamiliar usage of the closed suction system by healthcare professionals may be the leading cause of the contamination, resulting in the accumulation and countercurrent of the sputum into the 15-cm corrugated tube and Y-adapter, and some may even flow into the inspiratory and expiratory limbs through the breathing cycle.

A previous study found that when healthcare professionals performed the open-suction technique after disconnection from the ventilator system, the amount of suspended particles and bacterial concentration increased significantly, indicating an increased risk of exposure to biological aerosols [17]. Therefore, in order to maintain the safety of healthcare workers, it is recommended to avoid unnecessary ventilator system disconnection and the usage of a closed suction system to reduce the risk of bioaerosol exposure. The present study revealed that the bacterial concentration at the inspiratory limb of the reused ventilator system was significantly lower than that at the inspiratory limb of the disposable ventilator system, possibly because of a higher disconnection frequency in the reused ventilator system. As the reused ventilator system was disconnecting, the ventilator continued to deliver air, which might cause the dispersion of bacteria from the ventilator system into the air of the surrounding environment. In addition, as a disposable system with a high bacterial concentration is disconnected, the risk of exposure to biological aerosols for healthcare professionals increases apparently. Therefore, the importance of reused ventilator system sterilization should be addressed. The reused and disposable ventilator systems are recommended to avoid unnecessary disconnection of the ventilator system. When disconnection of the ventilator system is required, the healthcare professionals should wear protective masks for health and safety.

In the present study, four patients had no bacteria in their sputum culture, but contamination occurred in both ventilator systems. The source of these bacteria should be explored further in future studies. Additionally, no significant difference in the levels of human and environmental bacteria between the reused and disposable ventilator systems was found. Additional studies are warranted to clarify the possible sources of environmental bacteria in the ventilator system. Moreover, it is not necessary to change the ventilator circuit system routinely unless ventilator circuits appear visibly soiled or malfunction suggested by the AARC [9]. In this study, both the reused and disposable ventilation circuit systems had high bacterial detection rates and concentrations. The ventilator circuit systems whether should be changed more shorter period of time warrant further investigation.

It was difficult to enroll more patients for participation in this study. Changes in clinical conditions, such as sputum culture results showing multiple drug-resistant bacteria, and extubation from stable condition led to study withdrawal. The total number of patients in this study was 16, less than expected; however, there was still sufficient statistical power to yield statistical differences in the study results.

## Conclusions

A high rate of bacterial contamination was found at different locations in the reused and disposable ventilator systems, and also environmental bacterial species were identified in these systems. It is necessary to address the importance of sterilization of reused ventilator systems. We recommend that unnecessary disconnection of both types of ventilator systems during system operation should be avoided in order to promote the health and safety of healthcare personnel.

## Supporting information

S1 File Bacterial concentration and system disconnection frequency. (XLSX)
